# Magnetic Nanostructures as Emerging Therapeutic Tools to Boost Anti-Tumour Immunity

**DOI:** 10.3390/cancers13112735

**Published:** 2021-05-31

**Authors:** Stefano Persano, Pradip Das, Teresa Pellegrino

**Affiliations:** Nanomaterials for Biomedical Applications, Istituto Italiano di Tecnologia (IIT), Via Morego 30, 16163 Genova, Italy; pradip.das@iit.it

**Keywords:** magnetic nanostructures, surface chemistry, cancer immunotherapy, immune therapeutics, combinatorial immunotherapy, vaccines, immunogenic cell death

## Abstract

**Simple Summary:**

Immunotherapy represents an attractive therapeutic option for effective treatment of different forms of cancers. However, many patients exhibit primary or acquired resistance to conventional immunotherapies. In recent years, the encapsulation of immunotherapeutics into nanoparticles is emerging as a promising strategy to improve the responsiveness of immunologically “cold” tumors. Among the several types of nanoparticles explored for cancer immunotherapy, magnetic nanoparticles are particularly interesting since they act as carriers for immunotherapeutic agents and display self-adjuvanting properties. In addition, the ability of magnetic nanostructures to respond to an altering magnetic field (AMF) can be exploited to facilitate the movement of therapeutic-loaded nanoparticles at the desired site or promote localized heating for the thermal ablation of tumors. This review examines the peculiar properties of magnetic nanoparticles and how they can be harnessed for the design of innovative single or combinatorial cancer immunotherapies.

**Abstract:**

Cancer immunotherapy has shown remarkable results in various cancer types through a range of immunotherapeutic approaches, including chimeric antigen receptor-T cell (CAR-T) therapy, immune checkpoint blockade (ICB), and therapeutic vaccines. Despite the enormous potential of cancer immunotherapy, its application in various clinical settings has been limited by immune evasion and immune suppressive mechanisms occurring locally or systemically, low durable response rates, and severe side effects. In the last decades, the rapid advancement of nanotechnology has been aiming at the development of novel synthetic nanocarriers enabling precise and enhanced delivery of immunotherapeutics, while improving drug stability and effectiveness. Magnetic nanostructured formulations are particularly intriguing because of their easy surface functionalization, low cost, and robust manufacturing procedures, together with their suitability for the implementation of magnetically-guided and heat-based therapeutic strategies. Here, we summarize and discuss the unique features of magnetic-based nanostructures, which can be opportunely designed to potentiate classic immunotherapies, such as therapeutic vaccines, ICB, adoptive cell therapy (ACT), and in situ vaccination. Finally, we focus on how multifunctional magnetic delivery systems can facilitate the anti-tumour therapies relying on multiple immunotherapies and/or other therapeutic modalities. Combinatorial magnetic-based therapies are indeed offering the possibility to overcome current challenges in cancer immunotherapy.

## 1. Introduction

In the past years, a cumulative number of studies have highlighted the critical regulatory role of the immune system in tumour biology [1]. Indeed, it has been proven that the host’s immune system interacts with tumour cells throughout the process of cancer formation and progression, shaping the immunogenicity of tumours, either inhibiting or promoting tumour growth and development [2]. These findings have provided the basis for the development of novel cancer therapeutics; however, such complex mechanisms are still a matter of study and pertain to the medical breakthroughs started in the last decade, but which still hold great promise [3].

T cells have been shown to be major players in the generation of protective immunity and, as pointed out by Galon et al., the presence of tumour-infiltrating CD8^+^ T cells greatly influences the fate of a tumour [4]. Functional analyses of tumour-infiltrating T cells have contributed to a more detailed tumour stratification, which was found to better represent prognostic tools in the treatment of colorectal carcinoma than standard histopathological classifications [4,5,6].

**Figure 1:**
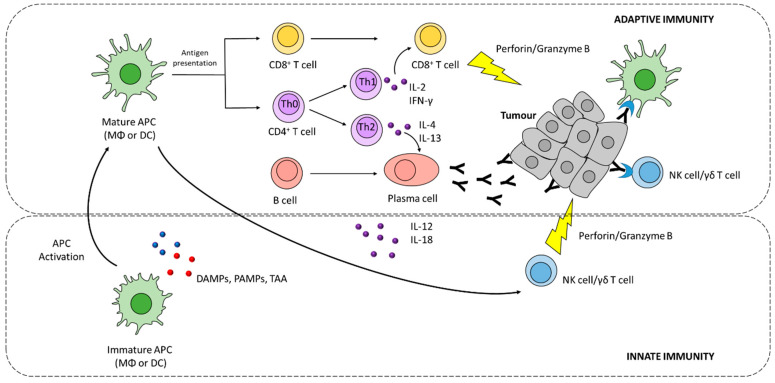
Overview of innate and adaptive anti-tumour immunity. Activated NK cells and γδ T cells can directly recognize and kill tumour cells through the release of perforin and granzyme B. Antigen-presenting cells (APCs), such as macrophages and dendritic cells, represent the main link between innate and adaptive immunity. Resting APCs can be activated by DAMPs and PAMPs, and then migrate to the secondary lymphoid organs where they present antigens and activate lymphocytes (CD8^+^ and CD4^+^ T cells, B-cells). CD4^+^ T cells primarily provide help for B lymphocytes and CD8^+^ T cells, whereas most CD8^+^ T cells exhibit cytotoxicity toward tumour cells. On the other hand, B cells are the source of antibodies directed against the tumour, which contribute to tumour recognition and antibody-dependent cell cytotoxicity (ADCC).

Although the activation of antigen-specific CD8^+^ T cells is considered a key step for an effective anti-tumour response, it often fails to eradicate cancer cells without a proper activation of the innate immune system [7]. Innate immune cells, such as natural killer (NK) cells, γδ T cells, and macrophages, can recognize and kill tumour cells [8]. Their activation occurs in response to danger signals released by stressed cells. These signals include pathogen-associated molecular patterns (PAMPs) and damage-associated molecular patterns (DAMPs), which are recognized by pattern recognition receptors (PRRs) expressed by immune cells, such as macrophages, dendritic cells, and NK cells. (Figure 1) [9]. Particularly relevant in tumour control is the role of NK cells, which together with T cells play a complementary function in contrasting tumour growth and propagation [10]. Indeed, NK cells can recognize cells with reduced or absent expression of major histocompatibility complex (MHC) class I molecules, thus ensuring the elimination of cancer cells that evade T cell-mediated killing [10].

Besides exerting its effector activity, innate immune cells have a pivotal role in directing and shaping the type and strength of anti-tumour adaptive immune responses, through the release of pro-inflammatory signalling molecules such as interferon gamma (IFN-γ) and interleukin-12 (IL-12) (Figure 1) [11,12].

Adaptive immunity, involving CD8^+^, CD4^+^T cells, and B cells, drives a tumour-specific response aimed at eradicating tumour cells, and contributes to the development of an immunological memory potentially protecting from tumour recurrence.

A series of events are required for the generation of antigen-specific anti-tumour responses, starting with the release of tumour antigens that are taken up by antigen-presenting cells (APCs), such as macrophages (MΦ) and dendritic cells (DCs), and processed into peptides [13,14]. Processed epitopes are loaded onto MHC I or II molecules for cross-presentation and presentation to CD8^+^ and CD4^+^ T cells, respectively (Figure 1) [14]. In order to induce effective T cell responses, antigen presentation must be supported by costimulatory signals induced by innate immune cells, such as pro-inflammatory cytokines and costimulatory ligands [15]. Furthermore, tumour antigens can also promote B cell activation by binding B cell receptor (BCR) [16].

Effector T cells must infiltrate tumour tissues where they recognize tumour antigens presented via MHCI, and selectively kill tumour cells. Tumour cell killing further promotes the release of tumour antigens, which can serve to prime additional T cell responses [17]. CD4^+^ T cells (or T helper cells, Th cells) regulate both cytotoxic cellular immune responses and B cell-dependent antibody production. Naïve CD4^+^T cells (Th type 0, Th0) can be activated by the encounter with a tumour antigen via peptide/MHC class II TCR and differentiate into Th type 1 (Th1) and Th type 2 (Th2) cells, depending on the intensity of stimulation and presence of certain cytokines and other factors (Figure 1) [17]. Th1 cells are characterized by the production and release of IFN-γ, which support tumour cytotoxicity synergistically with TNF-α (Figure 1) [17]. Th2 cells are mostly involved in the humoral immune response by promoting germinal centre formation and B cell function, through the secretion of IL-4 and IL-13 (Figure 1) [17]. After the immune response is completed, most effector Th cells will undergo apoptosis, while the remaining cells contribute to the CD4^+^T cell memory pool [18].

Despite the sophisticated and concerted anti-tumour immune response, the protective immunity of cancer patients often fails, as tumour cells have developed multiple mechanisms to evade immune surveillance [19]. These mechanisms are ascribable to (i) reduced immune recognition, either by the loss of immunogenic tumour antigens or by the downregulation of antigen-presenting molecules; (ii) increased tumour cell resistance to cytotoxic pathways; (iii) induction of an immunosuppressive tumour microenvironment, through the expression of immunoregulatory molecules (programmed death-ligand 1, cytotoxic T-lymphocyte antigen-4); and (iv) the recruitment of regulatory cells, including myeloid-derived suppressor cells (MDSCs), regulatory T cells (Tregs), and tumour-associated macrophages (TAMs), that will secrete immunosuppressive cytokines, such as interleukin 10 and transforming growth factor-beta (TGF-β) [2,19].

The identification of the immune-evading mechanisms of tumours is resulting in novel therapeutic strategies aimed at reversing tumour immune evasion. Particular interest is given to the development of strategies that can enhance the recognition of tumour cells by the immune system, such as therapeutic vaccines, adaptive cell therapy, and immunogenic cell death (ICD)-inducing treatments [5,20]. Other approaches are aimed at potentiating anti-tumour responses through the employment of immunotherapeutics, targeting immune checkpoint molecules (i.e., ICBs), and immunomodulators, such as immune adjuvants and cytokines, which, in turn, enhance cytotoxic T cell functions [5,20].

However, standard soluble immunotherapy has often failed to trigger effective cancer immune responses. This lack of effectiveness is due to an inadequate delivery of immunomodulators, as a consequence of their rapid degradation and elimination as free molecules. Likewise, DCs inappropriately uptake soluble vaccine antigens and adjuvants, resulting in an impaired antigen presentation and priming of anti-tumour immune responses [21,22,23].

To overcome the delivery limitations of soluble immunotherapies, nanoparticles have emerged as versatile vectors for the encapsulation, protection, and spatial–temporal-controlled delivery of antigens, adjuvants, and immunomodulators, while allowing, by controlling the structural parameters of the nanoparticles, to increase the uptake efficiency to targeted cells [23,24].

Magnetic nanoparticles (MNPs) represent an attractive class of nanomaterials due to their unique physical and chemical features that allow them to respond specifically to magnetic fields [25]. Among the magnetic class of materials, iron oxide-based nanoparticles are the only nanomaterials that have been approved by the Food and Drug Administration (FDA) for medical applications [26]. Magnetic nanomaterials are particular appealing for cancer immunotherapy due to their unique features, which include (i) the traceability of their signal by magnetic resonance imaging (MRI) or by magnetic particle imaging (MPI) techniques [27]; (ii) their exploitation as carriers to promote the accumulation and the efficient delivery of biotherapeutic compounds, such as genes and peptides, into a specific target cell or tissue; (iii) their ability to mediate the destruction of cancer cells through the production of a local thermo-ablative effect when exposed to an external alternating magnetic field, referred to as magnetic hyperthermia therapy (MHT) [25,26,27,28]; and (iv) their intrinsic immunomodulatory properties that can be harnessed to further promote or modulate the immune function.

Progress on the synthesis and functionalization procedures in the last few decades have enabled to obtain MNPs with very-well-controlled physicochemical features, including size, shape, crystallinity, charge, magnetic properties, and surface functionalities [25,28]. Furthermore, compared to nanoformulations conventionally applied for cancer immunotherapy, such as polymeric and lipid nanoparticles, MNPs can be easily synthesized with inexpensive procedures suitable for large-scale production [29].

All these features make MNPs suitable platforms for the development of combinatorial immunotherapies with enhanced therapeutic efficacy, by simultaneously tackling different tumour immune-escape mechanisms [25,28,30].

This review provides an overview of the recent advances in the use of MNP-based nanostructures to enhance the effectiveness of cancer immunotherapy. We highlight the impact of the physicochemical features and surface engineering of magnetic delivery platforms, and how they can be opportunely modified to potentiate therapeutic cancer vaccination, adoptive-cell therapy, immunomodulatory drugs, and ICD-inducing treatments. Finally, the article describes the use of magnetic nanosystems to enable the development of combinatorial therapeutic approaches for improving the efficacy of cancer immunotherapies.

## 2. Magnetic Nanomaterials for Cancer Immunotherapy: Synthesis and Properties

MNPs, thanks to their response to a magnetic field of a different nature, show unique advantages compared to other types of nanocarriers, which make them also promising for the field of cancer immunotherapy [25,28]. In particular, their unique capability as contrast agents in non-invasive molecular imaging techniques, such as MRI and MPI, can assist in the monitoring of the accumulation of magnetic nanoformulations at the target site [31]. Likewise, the utilization of MNPs as heat mediators in magnetic hyperthermia enable tumour ablation and the priming of anti-tumour immunity [32,33]. As the efficiency of MNPs as contrast agents as well as heat mediators depends on their physicochemical properties, the optimization of these properties is required for the synthesis of high-quality MNPs with a tunable size, shape, and composition.

MNPs usually have an overall hydrodynamic size smaller than 100 nm with a typical magnetic core size below 30 nm. Their magnetic properties can be tuned by the choice of size, shape, crystalline structure, and composition, among which iron oxides, such as magnetite (Fe_3_O_4_) and maghemite (γ-Fe_2_O_3_), or other mixed ferrites, such as zinc-ferrite (ZnFe_2_O_4_) or manganese-ferrite (MnFe_2_O_4_), are the most relevant for immune applications given the minimized toxicity of the Fe, Zn, and Mn ions of which these ferrites are made. Size is considered among the most important parameters affecting the magnetism of nanoparticles. Indeed, once the iron oxide nanoparticles become smaller than a critical size, they become superparamagnetic, exhibiting many desirable characteristics, such as no remanence and zero coercivity at room temperature, coupled with a high magnetic responsivity (susceptibility) and a reduced risk of self-agglomeration, since they exhibit their magnetic behaviour only when an external magnetic field is applied [25,28]. Moreover, magnetic properties can be fairly modulated by varying other physicochemical features related to surface structure and colloidal stability, or in other words, to the aggregation state of individual MNPs [25,28]. Indeed, the magnetic properties of iron oxide nanoparticles can be further redesigned by clustering a controlled number of individual superparamagnetic nanoparticles into superparamagnetic nanoparticle clusters, often termed magnetic nanobeads [34]. These clusters may also have a different arrangement, being, for instance, chain-like assemblies or 2D-clusters, which have peculiar magnetic features in MHT, MRI, and MPI.

**Figure 2:**
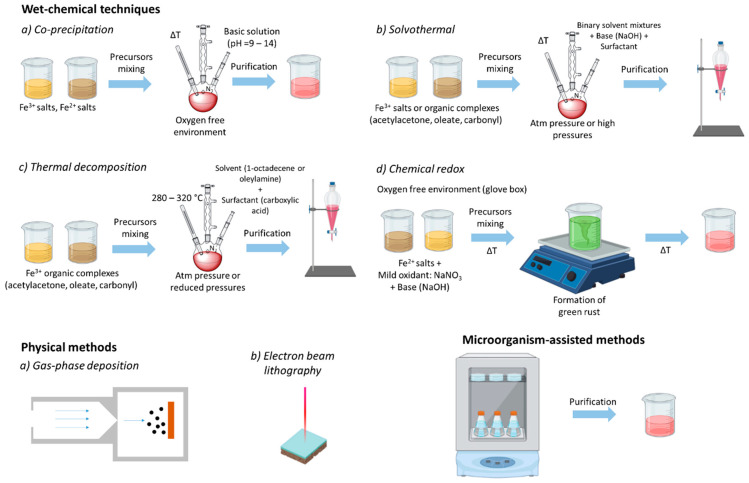
Different methods proposed for the synthesis of magnetic nanoparticles: wet chemical techniques (co-precipitation, solvothermal, thermal decomposition, chemical redox, etc.), physical methods (gas-phase deposition and electron beam lithography), and microorganism-assisted methods.

A wide range of methods have been reported for the preparation of high-quality MNPs, including wet chemical techniques (co-precipitation, solvothermal, thermal decomposition, sol-gel synthesis, microemulsion, and chemical redox), physical processes (gas-phase deposition and electron beam lithography), and bacterial and microorganism-based synthesis (Figure 2) [35,36,37]. Among these methods, co-precipitation, solvothermal, and thermal decomposition are the most commonly employed manufacturing processes. The first two methods represent easy and convenient nanofabrication approaches for a gram-scale MNP production, whereas the third method enables an unprecedented accurate control of the shape, size, composition, and polydispersity—at the milligram scale—of material production. Usually, wet chemical methods, such as the thermal decomposition method, involve the decomposition of precursors into liquid media, such as 1-octadecence, at a high temperature and in the presence of capping agents and surfactants, such as oleic acid [36]. During the synthesis, the reaction conditions, including temperature and pressure, play important roles in determining the morphology and size of the MNPs, and consequently their magnetic properties [36].

Depending on the preparation route, the as-synthesized MNP solubility may significantly differ. For instance, thermal decomposition and the solvothermal method can deliver MNPs soluble in non-aqueous media, as they are coated by alkylic surfactant molecules (such as oleic acid). On the contrary, MNPs prepared by the co-precipitation method are directly soluble in aqueous media, being coated by tiny polar molecules (such as sodium citrate). In both cases, the MNPs can be stabilized and functionalized by adding/replacing an outer layer of the shell coating, which can have multiple roles. First, it can serve as a stabilizing and protecting layer, slowing down the degradation of the magnetic core. Second, it can increase the stability in physiological media. Third, it can also introduce chemical groups feasible for the further functionalization of the MNPs with different biomolecules [28].

### 2.1. Optimization of the Physicochemical Parameters for Enhanced Magnetic Nanostructure-Based Cancer Immunotherapy

To optimize the effectiveness of MNP-based immunotherapy, key structural design considerations must be taken into account.

Early studies focused on nanoparticle delivery to tumours exploiting a mechanism known as the enhanced permeability and retention (EPR) effect [38]. Alternatively, the uptake could be further boosted by surface functionalization with tumour-targeting molecules [38]. Both passive and active tumour accumulation methods are affected by the MNP size and also by their surface chemistry. In particular, a size of less than 100 nm has been identified as optimal to ensure higher accumulation of iron oxide nanoparticles to tumours [38]. While these delivery strategies of nanoparticles directly to the tumour are becoming an increasingly appealing option for reshaping the tumour microenvironment, the design of novel nanosystems for cancer immunotherapy is also aimed to trigger tumour-specific responses by harnessing the natural tropism of nanoparticles towards secondary lymphoid organs (including spleen and lymph nodes), where T cell priming occurs. Delivery systems with hydrodynamic diameters of 10–100 nm and minimized interactions with the extracellular matrix (ECM) show improved diffusivity through the interstitium and therefore superior drainage to the lymphatic system when administered locally (intramuscularly or subcutaneously) [38]. For instance, lipidoid-stabilized iron oxide nanoparticles with a 30 nm core size had approximately 20-fold higher capacity to carry biomolecules such as antigens and adjuvants to lymph nodes via lymphatic drainage compared to smaller (10 nm) or larger (100 nm) nanoparticles [39].

Delivery platforms with a large size (>500 nm) lead to a prolonged retention at the injection site and are mostly taken up by local DCs, which after nanoparticle internalization will migrate to the draining lymph nodes [40]. Interestingly, a biodistribution study shows that transport through the lymphatic system results in an ~1000-fold increase in accumulation into local draining lymph nodes, which can substantially reduce off-target side effects and improve T cell priming [41]; thus, nanoparticles with a size around 30 nm may be preferred for lymph node targeting.

**Figure 3:**
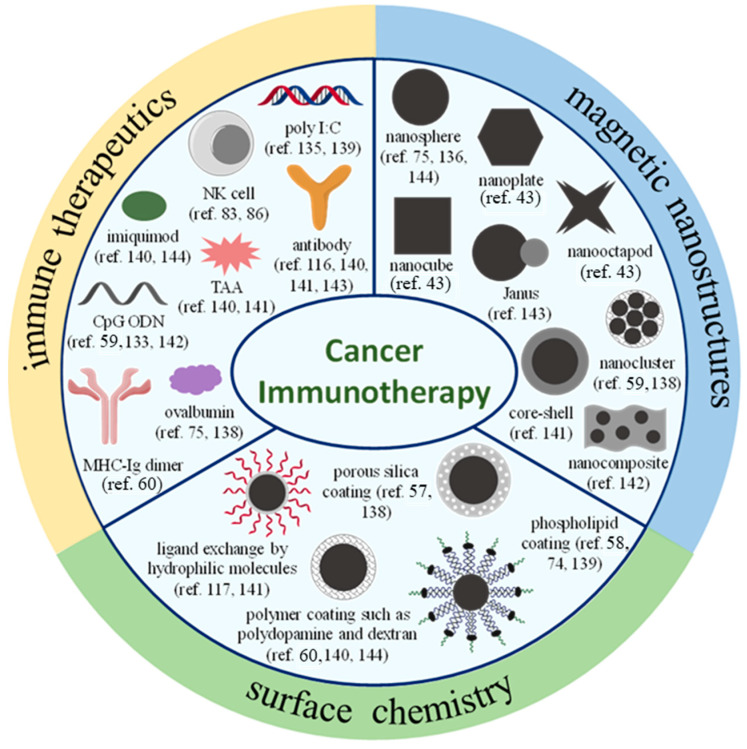
A schematic diagram summarizing the design and synthesis of magnetic nanostructure-based nanosystems for cancer immunotherapy.

Along with size, particle shape is another important parameter affecting the immunological response to nanoformulated immunotherapies [38]. The initially proposed nanoformulations were mainly spherical, but recent advances in nanofabrication have generated a wide range of other shapes (rods, prisms, cubes, stars, and discs) [42]. Therefore, various magnetic nanostructures, such as the nanosphere, nanocube, nanocluster, and nanocomposite, as shown in Figure 3, have been explored during the last decade in preclinical studies in order to develop highly effective nanomedicine-based cancer immunotherapies. Among the various morphologies of iron oxide nanoparticles, octapod- and plate-shaped nanoparticles with a similar aspect ratio and surface charge showed a higher immunomodulatory potential by inducing inflammasome activation [43]. The MNP’s shape also determines their distribution and uptake by immune cells. Generally, spherical nanoparticles’ internalization is favoured over non-spherical nanoparticles [44]. However, spherical nanoparticles diffuse less efficiently through the vascular wall than rod- and bar-shaped nanoparticles with a similar size range [44]. Therefore, morphological modulation of magnetic nanostructures is critical to determine their in vivo fate and their ability to target immune cells. Moreover, besides affecting their biodistribution and interaction with immune cells, the size, shape, and composition can also influence the intrinsic properties of MNPs, among which is their heating ability under an alternating magnetic field (AMF), expressed as the specific absorption rate (SAR) [45]. It has been reported that reshaping iron oxide nanoparticles from spheres to cubes markedly increases their heating performance [46]. In addition, controlled clustering of iron oxide nanocubes into nanoparticle assemblies that are anisotropic in their shape can preferentially increase the MNPs’ heating power [47].

### 2.2. Surface Engineering of Magnetic Nanostructures for Cancer Immunotherapy

The charge at the surface of MNP-based nanostructures, generating the interface between MNPs and the physiological environment, is also a crucial parameter to be optimized to ensure the desired therapeutic outcome [48,49]. Surface charge, in particular, is largely dictated by the coating materials and has a significant effect on the interaction with the immune system. Generally, local administration of positively charged MNPs promote a stronger immune response than nanoparticles having a net negative or neutral surface charge [50,51]. Though, cationic nanoparticles display reduced tissue penetration, probably due to the interaction with the negatively charged components of the ECM [52]. Consequently, positively charged nanoparticles are usually retained at the injection site, where they can be more easily taken up by local DCs, compared to neutral and anionic nanoparticles [53]. However, highly cationic nanocarriers are not appropriate for direct lymphatic transport and trafficking in the blood circulation, as they may induce haemolysis and platelet aggregation, resulting in premature antigen release, fast clearance, and high variability in the immune responses induced by these nanoformulations [54,55]. Contrarily, slightly negative-charged nanoparticles or neutral nanoparticles possess a superior circulation time and therefore may achieve enhanced tumour accumulation when systemically injected.

Besides surface charge, other surface physicochemical properties can affect tremendously the behaviour of MNPs in biological conditions, thus improving targeting efficiency, biocompatibility, therapeutic efficacy, stability, loading capacity, and efficiency [56]. After synthesis, most of the MNPs prepared by non-hydrolytic methods are capped by long hydrophobic chains that act as stabilizing agents, making them soluble in organic solvents [36]. Consequently, surface modification of these nanoparticles is firstly required to enable their water solubilisation, making them ready for any further modification. For nanoparticles produced by hydrolytic methods, charged capping molecules, such as citrate molecules, are usually exchanged with other spacer ligands, such as polyethylene glycol derivatives or dextran shells, which help to improve long-term colloidal stability in biological environments [35,36].

Surface modification has been also exploited to facilitate the loading of immunomodulators that can activate and/or boost the immune responses in patients [57,58]. The most common surface modification strategies, such as ligand exchange, porous silica, phospholipid, and polymer coating, have been extensively explored to facilitate loading of various immunotherapeutics, including TLR agonists and monoclonal antibodies onto MNPs through non-covalent or covalent interactions, taking advantage of the properties associated with the coating material (e.g., large pore size of the porous silica shell and large number of reactive functional groups of polymers) (Figure 3) [57,58,59,60].

A range of surface chemistry strategies also has been explored to facilitate multiple-drug loading. In this regard, the highly porous structures of mesoporous silica-coated ferumoxytol nanoparticles were capable to load both a checkpoint inhibitor (anti-PD-L1 antibody) and chemotherapeutic drug (cabazitaxel) for achieving an anti-tumoural synergistic effect against prostate cancer [57]. Likewise, surface modification of MNPs with a lipid shell enabled the co-encapsulation of the α-helix-antigen fusogenic peptide (α-AP) with indocyanine green (ICG), an imaging agent, leading to the development of a theragnostic nanoplatform (α-AP-fmNP) [58]. In the context of DC-based vaccines, α-AP-fmNP-loaded DCs were revealed to possess antigen presentation capability and their in vivo migration toward lymph nodes, as confirmed by imaging techniques, was dramatically enhanced upon application of magnetic force, thus preventing anergy and resulting in a significantly improved anti-tumour efficacy [58].

Recent studies have highlighted the intrinsic immunological properties of iron oxide nanoparticles that enable them to serve as immune adjuvants or immunomodulators [61]. In a pioneer study, iron oxide nanoparticles coated by carboxy-dextran were proven to activate the NF-κB pathway in macrophages, which plays important roles in inflammatory responses and immune activation/regulation, promoting M1 macrophage polarization [62]. The use of proper surface modification for modulating or enhancing the biointeractions of MNPs with immune cells was highlighted in a study employing commercially available, FDA-approved carbohydrate-coated superparamagnetic iron oxide nanoparticles (ferumoxytol) [63]. The authors demonstrated that the intravenously injected ferumoxytol nanoparticles could promote tumour regression as a consequence of the recruitment of pro-inflammatory M1-type macrophages at the tumour site. This interaction of the dextran-coated nanoparticles with macrophages was likely mediated by scavenger receptors [64]. Specifically, iron oxide nanoparticle recognition is realized through the binding between the positively charged collagen-like domain of the scavenger receptors and the dextran-coated MNPs. Furthermore, they showed that the type of coating introduced on the surface of the nanoparticles can have a strong effect on the nanoparticle–cell interaction. While 10 kDa dextran-coated MNPs were efficiently recognized and taken up by macrophages, the macrophage uptake was strongly impaired when nanoparticles functionalized with 20 kDa dextran or cross-linked dextran were employed, likely because the interacting groups of the polymer coating are sterically hidden in the latter two cases. Thus, it appears clear that the coating materials of the iron oxide nanoparticles have a significant influence on mediating the iron oxide nanoparticle’s immunomodulatory properties. Mulens et al., in this regard, reported that the polyethyleneimine (PEI)-coated superparamagnetic iron oxide nanoparticles induced Toll-like receptor 4 (TLR4) activation in both murine and human macrophages, with consequent upregulation of IL-12 production and surface expression of maturation markers such as CD40, CD80, and CD86, indicating M1 polarization [65]. Likewise, amino-polyvinyl alcohol-coated superparamagnetic iron oxide nanoparticles (a-PVA-SPION) led to increased IL-1β secretion in monocytes, as well as monocytes differentiation into macrophages [66]. Contrarily, treatment with different iron-based formulations (Venofer, Ferinject, and Ferrlecit) reduced the differentiation of monocytes into M1 macrophages and myeloid DCs [67], suggesting that the M1-polarization induced by PEI-iron oxide nanoparticles observed in the earlier studies could be influenced by the coating material and/or by the crystal phase of the MNPs (i.e., magnetite). Overall, these immunomodulatory properties could result in the induction of adaptive anti-tumour responses in vivo, even without the delivery of therapeutic payloads. Korangath et al. in this regard showed that systemic exposure to 100 nm-sized iron oxide nanoparticles coated by hydroxyethyl starch (i.e., bionized nanoferrite (BNF) nanoparticles) induced an immune response leading to CD8^+^ T cell infiltration, which was associated with tumour growth delay [68].

Besides the coating material, the chemical composition of the MNP core is another factor that influences the immunomodulatory properties of these nanoparticles. For instance, it has been shown that oppositely to hematite phase (Fe_2_O_3_) nanoparticles, magnetite (Fe_3_O_4_) iron oxide nanoparticles display a great capacity in promoting macrophage polarization from a pro-tumoural M2 into an anti-tumoural M1 profile [62,63].

## 3. Therapeutic Anticancer Vaccines

Therapeutic cancer vaccines are a form of immunotherapy that aims to stimulate the immune system to mount a response against tumour cells by exposing individuals to tumour-specific antigens.

Vaccines represent an optimal strategy to increase the immune responses towards tumours characterized by low immune infiltrates, by inducing T cell priming and expanding tumour-specific T cell responses [5]. However, as reviewed by Hu et al., traditional vaccination has failed to achieve clinical responses in most tumour settings but induces cellular immune responses sufficient to control tumour growth [69,70].

**Figure 4:**
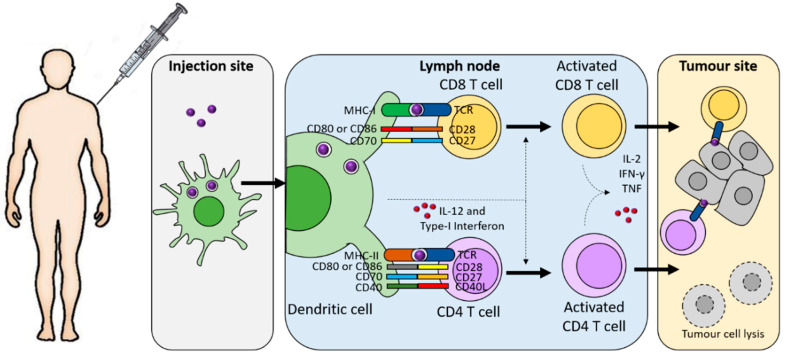
The tumour antigen presentation process induced upon vaccination. The antigen is rapidly taken up by antigen-presenting cells (APCs), such as dendritic cells, at the injection site. Subsequently, the APCs traffic towards the draining lymph nodes, where mature DCs present the antigen-derived peptide on MHC class I molecules and MHC class II molecules to CD8^+^ and CD4^+^ T cells, respectively. Several factors, such as costimulatory molecules (CD28, CD27, and CD40) and cytokines (IL-12 and Type-I interferon), concur in the activation of T cells. The generated activated antigen-specific T cell migrate to the tumour site and kill tumour cells through cytotoxicity and the production of effector cytokines, such as IFN-γ and tumour necrosis factor (TNF).

Effective therapeutic cancer vaccination involves first the identification of appropriate tumour antigens (or antigenic epitopes) and their subsequent administration to patients, employed as MHC-I-restricted peptides or nucleic acids (Figure 4) [71]. For the generation of effective and durable T cell responses, the selected antigens must be delivered to antigen-presenting cells, in particular to DCs either at the injection site, typically the dermis, or at lymphoid organs. DCs are key players in the generation of T cell anti-tumour immunity, as they mediate the processing of tumour antigens and their presentation to naïve CD8^+^ and CD4^+^ T cells in the draining lymph nodes. T cell activation has been shown to require the expression of co-stimulatory molecules and the secretion by DCs, after maturation, of pro-inflammatory cytokines, such as IL-12 [72,73]. Appropriate T cell priming will generate and expand the pool of tumour antigen-specific T-cells that, after trafficking to the tumour, will mediate tumour cell killing (Figure 4) [69]. Based on the above mechanism, four different aspects of cancer vaccines must be taken into consideration for the induction of anti-tumoural responses (Figure 4): (1) the selection of tumour antigens; (2) the choice of antigenic platform; (3) the incorporation of immune adjuvants to enhance the immune response; and (4) the antigen-delivery systems to carry the antigen to the DCs.

The administration of “naked” antigens in the form of protein, peptide, pDNA, or RNA (the latter two able to induce the expression of antigens) often fail to induce immunity due to the rapid degradation and clearance of the unprotected antigen/genetic material, and to the lack capability to enter the APCs. Sungsuwan and co-workers reported that iron oxide nanoparticles can overcome these delivery hurdles by preventing antigen degradation and improving its delivery to the target tissues [74]. In their study, the MNPs were individually coated with phospholipid-MUC1 peptide through hydrophobic–hydrophobic interactions. The synthesized MNPs exhibited a size around 35 nm, which enabled their accumulation in the axillar lymph nodes (draining lymph node), thus promoting their interaction with resident immune cells. In line with this, mice immunized with the MNP-based vaccine formulation generated strong humoral responses, as indicated by the increased production of anti-MUC1 IgG antibodies, measured by an enzyme-linked immunosorbent assay. The produced antibodies could recognize both murine and human tumour cells overexpressing MUC1, promoting tumour cell death through complement-mediated cytotoxicity (CDC). Recently, Luo and his collaborators developed ultra-small iron oxide nanoparticles functionalized with ovalbumin (OVA) as a model tumour antigen, with an overall size between 20 and 40 nm, that efficiently promotes DC maturation and consequent T cell priming at higher levels than “naked” OVA [75]. The OVA-coated MNPs upon subcutaneous administration not only inhibited the growth of subcutaneous and lung metastatic B16-OVA tumours, but were also successfully tested as a prophylactic vaccine, preventing the formation of subcutaneous and lung metastatic B16-OVA tumours. Interestingly, the authors showed that nanoparticles having a low surface density of MHC class I-restricted OVA-peptide promoted the induction of a more effective anti-tumour immune response than densely packed peptide nanosystems, likely attributed to a hindrance effect on the receptor–ligand interaction. This study underlines how the graft density of a surface-immobilized antigen on MNPs is another crucial parameter that should be considered in the design of iron oxide-based nanovaccines.

## 4. Adoptive Cell Therapy for Cancer Immunotherapy

Adoptive cell therapies (ACT) involve the isolation, subsequent expansion/manipulation, and ultimate reinfusion of lymphocytes (i.e., T cells, NK cells, and NKT cells) into patients, in an attempt to overcome inadequate T-cell priming, potentiating the patient’s immune response by providing lymphocytes with cytotoxic activity [76,77,78].

**Figure 5:**
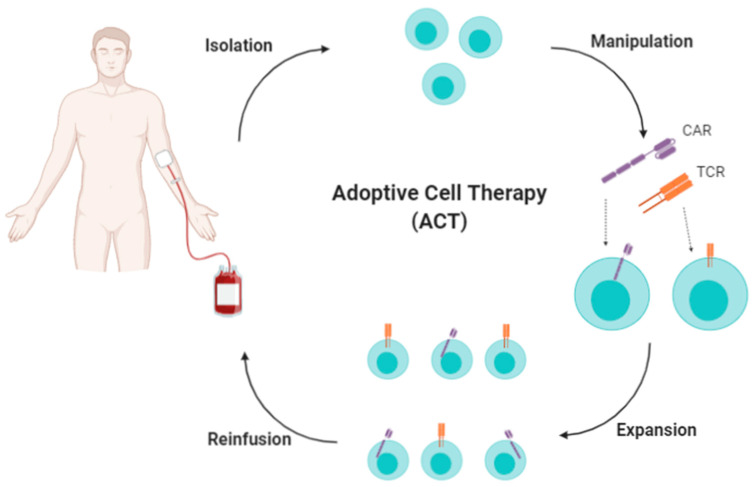
Overview of adoptive cell therapy in the clinic. Patient’s T cells or NK cells are harvested and subsequently manipulated ex-vivo, to enable them to identify and eliminate cancer cells. The activated T cells can be then genetically reprogrammed by transduction with a construct encoding CAR or TCR, and then the cells are further expanded. Finally, the cells are reinfused into patients.

Strategies employing ACT are classified into three methods, as follows (Figure 5): (1) isolation, expansion, and reinfusion of tumour-infiltrating lymphocytes (TILs) to produce a monoclonal population of tumour-specific T cells; (2) expansion of antigen-specific peripheral blood lymphocytes (PBLs), generating a polyclonal population of tumour-specific T cells; and (3) isolation and genetic modification of PBLs to confer tumour-specific antigen recognition in a population of T cells, typically, chimeric antigen receptors (CARs) targeting a tumour cell surface molecule (Figure 5) [78,79]. This last method has shown several potential advantages over conventional therapies, including specificity, a high success rate, and long-lasting effects, particularly in haematological malignancies. However, several factors limit the efficacy of ACT, particularly for most solid tumours [76,77,80]. It has been shown, for instance, that reinfused T cells do not last for extended periods of time in vivo and they might not reach the tumour or tumour draining lymph node to exert their cytotoxic function [76,80]. Indeed, a recent study revealed, in the context of a melanoma, failed trafficking into tumours of tumour-specific effector T cells that had been adoptively transferred [81]. Additionally, there are safety concerns associated with their use, as CARs are typically transduced into T cells using randomly integrating viral vectors that may lead to oncogenic transformation, variable expression levels, and transcriptional silencing [5].

Notably, the standard procedure for manufacturing autologous T cell-based therapies involves the use of superparamagnetic polymer microbeads (Dynabeads^TM^) covalently linked with anti-CD3 and anti-CD28 antibodies to provide the primary and co-stimulatory signals needed to activate and expand T-cells in a manner that partially mimics stimulation by antigen-presenting cells [82].

MNPs may be also employed at other stages of ACT therapies to overcome current limitations, for instance, by improving their tumour accumulation. Different studies showed that 3-aminopropyl-triethoxysilane (APS)-coated MNPs can be attached on the surface of effector T cells and NK cells, as confirmed by TEM analysis, without affecting their main functions (i.e., degranulation capacity, cytotoxic action on target cells, IFN-γ production, and chemotaxis) [83,84]. Furthermore, in a proof-of-concept study, it was reported that, by using an external magnetic field, it was possible to enhance the accumulation of the reinfused cytotoxic cells at the tumour site and their retention in the tumour-draining lymph nodes, thus promoting their anti-tumour effects [83,84,85]. Despite these encouraging preclinical results, the clinical feasibility of this approach remains to be investigated and confirmed, as it is still a challenge to limit the application of the magnetic field gradient only to the area that needs to be treated.

CAR-cell therapy is currently one of the most promising approaches for cancer treatment. Nonetheless, the development of CAR-cell therapies is challenging due to the generally low-efficient transfection of T cells and NK cells when employing lipofection reagents [86]. Magnetic nanostructures may find new applications by improving the transfection efficiency of the lymphocytes. Polydopamine (PDA)-coated MNPs functionalized with polyethyleneimine (PEI), for instance, were proven to efficiently deliver genetic materials and induce the expression of EGFR targeting chimeric antigen receptors (EGFR-CARs) on the NK cell surface, which improved the cells’ anti-cancer cytotoxic effect both in vitro and in vivo [86]. The authors showed that the employed multifunctional nanoparticles did not display any significant toxicity in vitro up to a concentration of 16 µg/mL (referred as iron concentration), although at higher amounts (64 µg/mL) they displayed a 4–5 times higher cytotoxicity towards NK cells, possibly due to the increasing amount of the non-biodegradable PEI [86].

The therapeutic potential of ACT immunotherapy is influenced by the capability of the lymphocytes to traffic towards tumours, and novel tracking techniques for monitoring the biodistribution of the transferred lymphocytes could potentially accelerate its development. Currently, there is still a lack of diagnostic tools to accurately predict and evaluate the therapeutic outcomes of ACT and how this is determined by their in vivo biodistribution, persistence, and proliferation [87]. While the radiolabelling of immune cells with ^18^F-FDG, ^111^In-oxine, and ^11^C-methionine has been a standard technique for years, new non-invasive methods with improved sensitivity could allow higher detection efficiency, while preserving cell activity and integrity. Li et al. labelled NK cells with heparin–protamine–ferumoxytol (HPF) nanocomplexes and follow their biodistribution within liver tumours upon intraperitoneal injection by MRI [88]. The uptake of HPF by NK cells upon incubation with different amounts of nanoparticles was confirmed by TEM and quantified using Prussian blue assay (0 μg/mL HPF = 0% (PBS control), 25 μg/mL HPF = 89 ± 3%, 50 μg/mL HPF = 92 ± 4%, and 100 μg/mL HPF = 97 ± 5%). The authors showed that there were no significant differences in the viability of labelled and unlabelled NK cells up to 50 μg/mL HPF labelling. This study demonstrated the feasibility to track by MRI the biodistribution of MNP-labelled NK cells.

MPI is a novel imaging technique that enables monitoring the biodistribution of SPION tracers, with no tissue background and a signal intensity proportional to the amount of SPIONs and magnetic field strength. Unlike MRI, the signal generated in MPI is not affected by the surrounding tissues and has better tissue penetration. In addition, this technique has demonstrated to be safe since it does not use ionizing radiation, and SPIO tracers break down in vivo and the released iron can be processed and metabolized by the body as iron cofactors (i.e., heme, iron–sulphur clusters, and simple iron ions). On this matter, Rivera-Rodriguez et al. performed a proof-of-principle study, demonstrating the potential of MPI to track lymphocytes by labelling them ex-vivo with a commercially available MR tracer (ferucarbotran) [27]. The nanoparticles were taken-up by the T cells and localized at the cytosolic compartment, as confirmed by Prussian blue staining. The labelling of T cells with this nanoparticle did not affect their viability and effector functions, as they were capable of producing IFN-γ and induce target cell killing. Upon systemic administration of ferucarbotan-labelled T cells (10^7^ cells in 100 μL), MPI allowed the tracking of T cell biodistribution, starting at time zero. After an initial accumulation in the lungs, the cells redistributed to other major organs, such as the liver and spleen. On the contrary, fluorescence imaging failed to detect T cells in vivo, mainly due to the attenuation of the fluorescence signal by tissue/hair after imaging for epifluorescence using an IVIS system. The above suggests that MPI represents an attractive technique to track immune cells and provides a unique insight into the fate of these cells following ACT to accelerate the development of novel cancer treatments.

Moreover, MPI is an emerging imaging technique that enables quantitative detection of iron oxide tracers in specific regions. Unlike MRI, where iron oxide nanoparticles are used to enhance the contrast of MR images by promoting relaxation of the surrounding water, MPI uses SPIONs as tracers to study the distribution in vivo [89].

## 5. Immunogenic Cell Death-Inducers for Anti-Tumour In Situ Vaccination

In recent years, many studies have demonstrated that dying cancer cells have the ability to elicit an immune response through the release or exposure of immunostimulatory DAMPs, resulting in T cell activation and proliferation, eventually culminating in eradication of the tumour [30,90,91].

**Figure 6:**
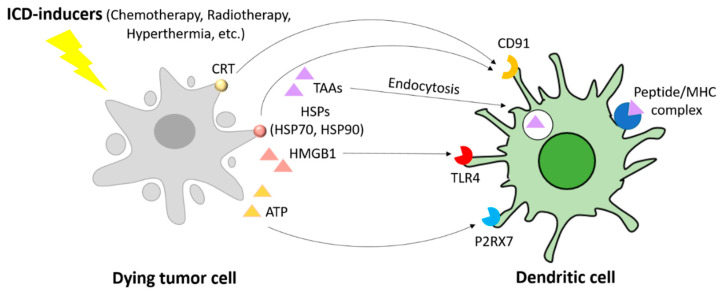
Schematic representation of the molecular mechanism occurring during immunogenic cell death process.

Immunogenic cell death (ICD)-mediated immune priming and activation is characterized by the induction of a distinct cascade of molecular events, including (i) the relocation on the plasma membrane of the endoplasmic reticulum (ER)-resident chaperone calreticulin (CRT) and the exposition of heat-shock protein 70 (HSP70) and HSP90, which together act as “eat me” signals upon binding to their transmembrane receptor CD91 (also known as LRP1) on immature DCs and macrophages, promoting phagocytosis [90,91,92,93]; (ii) the secretion of adenosine-5′-triphosphate (ATP) that will bind to the purinergic receptor P2RX7 expressed on DCs, leading to their recruitment at the tumour site [90,91,92,93]; (iii) the activation of a cancer cell-intrinsic type I IFN response and consequent secretion of CXC-chemokine ligand 10 (CXCL10), stimulating T cell recruitment; and (iv) the release of the non-histone chromatin-binding protein high-mobility group box 1 (HMGB1) into the extracellular environment that will bind to toll-like receptor 4 (TLR4) on DCs to promote maturation, antigen processing, and presentation (Figure 6) [30,90,91].

Overall, the binding of these DAMPs to cognate receptors on the surface of myeloid or lymphoid cells will favour the uptake of tumour cell debris by DCs and macrophages, in the context of robust immunostimulatory signals, leading to the priming of an adaptive immune response involving both αβ and γδ T cells and the establishment of immunological memory. As such, ICD-inducers have emerged as an in situ vaccination strategy, overcoming the limitations commonly associated with conventional vaccines. In addition, the response induced by ICD has the potential to eradicate malignant cells that survive chemotherapy via an IFN-γ-dependent mechanism, thus making it an attractive strategy for the development of combinatorial therapies [90,93].

Since its initial discovery, different ICD-inducing agents have been identified, such as certain types of chemotherapeutics, radiotherapy, hyperthermia, photodynamic therapy, some immune adjuvants, oncolytic viruses, and cytotoxic peptides [93,94,95,96]. The ability to induce directly or indirectly ER stress represents a common prerequisite of ICD-inducers. However, many cancers develop different mechanisms to prevent ICD-induced immune responses, for instance, via evasion of phagocytosis through upregulation of “don’t eat me” signals (i.e., CD47) or the expression of ectonucleotidases (i.e., CD39 and CD73) that hydrolyse extracellular ATP to adenosine [97,98].

Already in 1998, Yanase et al. reported that MHT with MNP-based cationic liposomes not only promote cancer cell elimination but can also induce systemic anti-tumour immunity, consequently inhibiting the growth of distant tumours (abscopal effect) [99]. After this early insight, multiple studies have provided evidence that local MHT can convert the tumour environment directly into an in situ vaccine [100]. Deeper studies have revealed that heat-stressed cancer cells release or expose on their surface DAMPs molecules that stimulate anti-tumour immune responses. Toraya-Brown and co-workers, in this regard, exploited commercially available starch-coated magnetite nanoparticles known as BNF of 100 nm diameter and applied an external alternating magnetic field (167.5 kHz, 45–55 mT) to heat B16 melanoma primary tumours to 43 °C for 30 min [101]. The treatment delayed tumour growth in both the treated primary tumour side and the contralateral side in a CD8^+^ T-cell-dependent manner. Remarkably, this strategy enabled the generation of a tumour-specific immunological memory that protected mice from B16 melanoma tumour re-challenge. The authors showed that tumour-free mice re-challenged with B16 cells did not grew tumours, while mice re-challenged with unrelated Lewis carcinoma cells developed tumours. Interestingly, this study also demonstrates that the heating temperature is a critical parameter to be optimised for the generation of anti-tumour immunity. Indeed, protection against tumour re-challenge occurred when tumours were heated at 43 °C, while it failed when tumours were heated at 45 °C, thus indicating that this temperature is not optimal for the priming of adaptive immune responses and the establishment of an immunological memory.

Moreover, local hyperthermia at 42 °C also increases tumour vasculature permeability [102,103], which together with enhancing drug delivery to the tumour site may also facilitate immune cell trafficking between tumours and lymphoid organs [104].

In addition to its effects on adaptive anti-tumour responses by inducing the release of tumour antigens, cytokines, and chemokines, temperature increases can also upregulate the tumour cells’ expression of stress ligands, such as major histocompatibility complex (MHC) class I–related chain A (MICA) and UL16-binding proteins (ULBP) 1, 2, and 3, all of them natural killer group 2, member D (NKG2D) ligands, making them more susceptible to lysis by NK cells, NKT cells, and γδ T cells [105,106], which may be particularly relevant in the treatment of tumours that had evaded CD8^+^ T cell-mediated killing. Although this phenomenon has been described in conventional hyperthermia, it has yet to be fully characterised in the context of MHT [107]. MHT being more spatially controlled and displaying a localized site-specific cytotoxicity, as shown in the pre-clinical settings, it would be of interest to see if this effect is partially mediated by sensitizing tumour cells to the killing action exerted by NK cells and NKT cells. Future studies dissecting the immunological role of MHT and the optimal therapeutic temperature range for maximizing anti-tumour immune responses may be particularly relevant for the field.

## 6. Immunotherapy Targeting Immune Checkpoint Molecules

The increased understanding of the complex network of immune interactions and the cancer cell antigens associated with tumours has led to the implementation of monoclonal antibodies-based treatments for the targeting of specific key pathways in the cancer immunity cycle [108]. Studies highlighting the role of cytotoxic T lymphocyte antigen-4 (CTLA-4) in blocking CD28 costimulatory signalling, controlling early stages of T cell activation, led to the development of monoclonal antibodies to target CTLA-4 to unleash cytotoxic T cell function [109]. Similarly, the identification that tumour cells overexpress immune checkpoint molecules, such as PD-L-1, on their surface in order to deactivate T cells and evade immunogenic cell death, has led to the implementation ofanti-PD-1 and anti-PD-L-1 immune checkpoint inhibitor therapies that are aimed at preventing tumour cell evasion by interfering with T cell suppression signals [110]. Checkpoint inhibitors are particularly efficient at unleashing pre-existing anti-tumour immune responses and this type of therapy is considered among the most successful forms of cancer immunotherapy [111]. Drugs blocking these pathways are currently utilized for a wide variety of malignancies and have demonstrated durable clinical activities in a subset of cancer patients [111,112]. This approach is rapidly extending beyond CTLA-4 and PD-1/PD-L1. New checkpoint inhibitory receptors are under investigation, and several drugs targeting these pathways are being investigated [108]. Despite this, not all patients have shown improved objective responses and survival upon checkpoint inhibitor therapies [112]. Indeed, significant heterogeneity in the responses has been reported in clinical studies, where immunological aspects of the tumour and the host are believed to play a key role in determining the response rate [112,113]. Additionally, there are safety concerns associated with their use, as their activity is not limited to the local tumour microenvironment. Systemic administration of anti-CTLA-4 antibodies, for instance, tends to activate self-reactive T cells systemically in cancer patients, thus greatly increasing its toxicity, due to an over-activation of the immune system, and limiting its use [114].

Engineered nanoparticles have emerged as a useful tool for the delivery of monoclonal antibodies directed against immune checkpoints at the desired site, increasing their therapeutic index, and MNPs, in particular, may promote a more precise tumour accumulation by means of magnetic-guided strategies [115]. For this purpose, Chiang et al. reported the used of superparamagnetic iron oxide nanoparticles conjugated with anti-PD-L1 and anti-CD3/anti-CD28 to enhance tumour-targeted delivery via magnetic navigation by means of an external magnetic field of 0.22 T applied to the tumour site of 4T1 tumour-bearing mice (as described in detail in Section 8.1). This strategy enabled an in situ expansion of tumour-infiltrating immune cells, minimizing systemic distribution of antibodies, and thus guaranteeing an improved therapeutic efficacy compared to soluble antibodies [116]. Luo et al. designed a magnetic polyplex consisting of positively charged folic acid functionalized PEI-MNPs with negatively charged small interfering RNA (siRNA) against PD-L1 (siPDL1) to block PD-L1/PD-1 interaction at the tumour site [117]. The authors showed that human gastric cancer cells transfected with siRNA-loaded MNPs exhibited effective PD-L1 knockdown, resulting in enhanced T cell activation. By targeting gastric cancer cells, the authors selectively reduced the expression of PD-L1 on the tumour microenvironment, thus preventing the generation of adverse effects associated with the broad blockage of the PD-L1/PD-1 interaction [117].

## 7. Immunomodulators to Enhance Anti-Tumour Immune Response

The lack of adequate innate immune responses represents the bottle neck to the activation of an effective adaptive anti-tumour response. For this reason, the administration of treatments addressed to reinvigorate anti-tumour functions without directly targeting tumour cells, have also gained attention in the context of excluded and immunosuppressed tumours, promoting the infiltration and function of immune effectors. These therapies involve the delivery of cytokines/chemokines or immune adjuvants that act as immunomodulators. Cytokines were introduced into the clinic almost 30 years ago [118]. Three main types of cytokines have been employed for immunotherapy: interferons, interleukins, and granulocyte–macrophage colony-stimulating factor (GM-CSF) [119]. Interferons, normally produced by immune cells in response to microbial pathogens, induce the maturation of NK cells, lymphocytes, and DCs, promoting antigen processing and presentation [120,121,122,123]. Interleukins, such as IL-2 or IL-15, stimulate the activity and growth of CD4^+^ T, CD8^+^ T cells, and NK cells [124,125,126,127]. GM-CSF, on the other hand, improves immune responses by supporting DC differentiation and thus by promoting T cell survival [128,129].

PRR agonists represent another class of immunomodulators, due to their ability to impact on multiple immune mechanisms, such as phagocytosis and antigen presentation. PRRs are activated upon release of “danger signals” by pathogens (e.g., viral DNA and bacterial proteins) or by upregulation of stress-associated molecules (e.g., HMGB1 and CRT) [130]. Natural endo/exogenous or synthetic PRRs agonists, such as Toll-like receptor (TLR) ligands, retinoic acid-inducible gene-I (RIG-I)-like receptors (RLRs) ligands, and stimulator of interferon genes (STING) agonists, have been applied in several preclinical and clinical studies for cancer treatment [131,132]. Despite their tremendous immunogenicity and therapeutic potential, the clinical use of immunomodulators has been often limited due to safety concerns related to their high toxicity as a result of systemic exposure, leading to an uncontrolled activation of the host immune system. The use of nanocarriers can potentially facilitate a precise delivery of immunomodulatory molecules, thus reducing the collateral effect caused by their off-target accumulation. Additionally, nanocarriers can improve their stability and enhance their uptake by target immune cells (e.g., APCs) [23]. White and co-workers showed that targeted magnetic delivery can be extended to immune cells, allowing the loading of cells with iron oxide nanoparticles functionalized with CpG oligonucleotides. MNPs were exploited to simultaneously promote the activation of immune cells and to magnetically control their trafficking towards the brain by an external magnetic field, thus ensuring a localized anti-tumour response [133,134].

In a different study, the use of amino-modified ferumoxytol was proposed for intratumoral delivery of TLR3-agonist poly(I:C) [120]. The local injection of adjuvant-loaded nanoparticles induced enhancement of macrophage-mediated tumoricidal activity against both subcutaneous and non-treated pulmonary metastatic tumours, resulting in melanoma regression [135]. MNPs can be further formulated to introduce nucleic acid sequences encoding immunostimulatory proteins, such as cytokines, into the target cells and stimulate the desirable immune responses in a more targeted approach [136]. For instance, superparamagnetic iron oxide nanoparticles coated with a double layer of polyacrylic acid (PAA) and PEI (SPIONs-PAA-PEI) were used to enable plasmid DNA binding onto the surface of nanoparticles by ionic interactions, and proved to be safe and effective to transfect a plasmid DNA encoding IL-12 into murine mammary adenocarcinoma tumours, resulting in an effective and localized anti-tumour effect [136].

## 8. Combinatorial Approaches to Potentiate Cancer Immunotherapy

The clinical successes achieved with the use of cancer immunotherapy mostly based on checkpoint inhibitors have profoundly changed the treatment of several malignancies [137]. However, there are still many challenges that need to be addressed in order to exploit the full potential of immunotherapy and improve the overall response rates in patients, as tumour cells develop multiple mechanisms to escape immune recognition and immune cell killing. As such, combination immunotherapy is emerging as a strategy to treat cancer; yet, effective synergism with enhanced safety is still under investigation. Indeed, the combination of multiple therapeutics frequently appears to induce stronger toxicity, potentially limiting their clinical implementation.

Several MNP-based platforms have been reported to facilitate the development of combinatorial treatments aiming at merging multiple immunotherapeutic approaches together (combinatorial immunotherapy) or to combine cancer immunotherapy with standard-of-care therapies, including chemotherapy or hyperthermal therapy (multimodal treatments), that are being evaluated in preclinical settings and have displayed promising results in enhancing the therapeutic effect of single-agent immunotherapy and potentially reducing the toxicity of combinatorial immunotherapies (Table 1).

### 8.1. Combinatorial Immunotherapies

**Figure 7:**
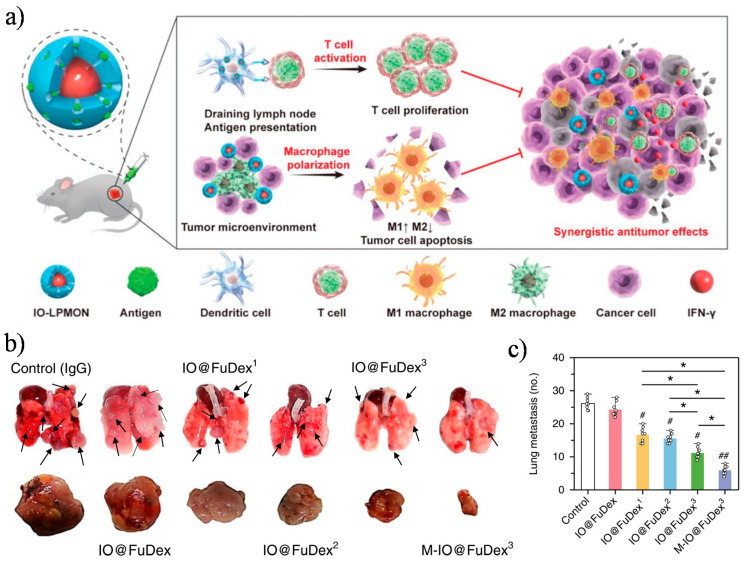
(**a**) Schematic representation of iron oxide-embedded, large-pore mesoporous organosilica nanospheres (IO-LPMONs) loaded with OVA protein. OVA-loaded IO-LPMONs are taken up by dendritic cells, which upon activation migrate to the draining lymph nodes and trigger the initiation of specific immune responses. Simultaneously, the nanovector acts not only as a carrier, but also as an immunomodulator, promoting the repolarization of TAMs from an M2 to M1 profile. (**b**) The photographs of dissected breast primary tumours and metastatic lungs from mice treated with the various IO@FuDex nanoformulations (IO©FuDex, no antidobies; IO@FuDex^1^, only anti-CD3/CD28 antibodies; IO©FuDex^2^, only anti-PD-L1 antibody; IO@FuDex^3^, anti-CD3/CD28 and anti-PD-L1 antibodies; and M-IO@FuDex^3^, anti-CD3/CD28, and anti-PD-L1 antibodies + magnetic navigation). The arrows point the metastatic nodules in the lungs. (**c**) Quantification of the metastatic nodules confirm that MNPs can support the synergistic anti-tumour effect of checkpoint inhibitors and T cell activators, and that of the therapeutic efficacy of IO@FuDex3 by promoting specific localization of the therapeutics with magnetic navigation (**b**). Reproduced with permission from References [116,138] (Copyright 2019 Wiley-VCH and Copyright 2018 Nature Publishing Group).

A promising approach to enhance the effectiveness of cancer immunotherapy involves the combination of treatments addressed to generate or expand antigen-specific cytotoxic immune responses, for instance through cancer vaccines, with therapeutic approaches designed to balance the immune suppressive TME. The combined effects of these approaches can be further improved using magnetic nanocarriers, enabling the co-loading of antigens and adjuvants and boosting the tumour or lymph node targeting selectivity. For example, the stimulation of T cell response through the delivery of an antigen (OVA), with the simultaneous repolarization of tumour-associated macrophages (TAMs), was achieved using magnetic core–shell nanospheres (IO-LPMONs) composed of an iron oxide (IO) core and a mesoporous organosilica shell with large pores (6.3 nm diameter), which allowed a high encapsulation efficiency of OVA and its delivery to DCs [138]. The formulation was proven to stimulate the maturation of DCs and consequently the expansion of both CD4^+^ and CD8^+^ OVA-specific T cells, which resulted in a strong T cell immunity against tumours (Figure 7a). In addition, it was also demonstrated in this work that the repolarization of TAMs from an immunosuppressive M2 phenotype to tumour suppressing M1 phenotype was achieved due to the intrinsic adjuvant property of iron oxide nanoclusters. The synergistic effects of T cell activation together with macrophage repolarization demonstrated an enhanced therapeutic efficacy, inhibiting tumour growth.

In another strategy, iron oxide nanoparticles were modified with a checkpoint inhibitor (anti-PD-L1 antibody) and anti-CD3/CD28 antibodies providing activating signals to T cells, with the aim to overcome the immunosuppressive tumour microenvironment and promote the anti-tumoural activity of tumour-infiltrating lymphocytes [116]. Anti-CD3, anti-CD28, and anti-PD-L1 antibodies were conjugated onto the surface of fucoidan dextran-coated iron oxide nanoparticles (IO@FuDex) by using a reductive amination. The obtained multifunctional magnetic nanoparticles (IO@FuDex^3^) were intravenously administrated into 4T1 mammary carcinoma-bearing mice. To minimize the undesired off-target accumulation of the antibodies and to achieve in situ expansion of tumour-infiltrating T cells, an external neodymium magnet of 0.22 T was applied at the tumour site for 4 h for three consecutive days (4 h/day). The authors showed that the field gradient of more than 10 T/m at the distance of 2 cm from the applied magnet significantly favoured the accumulation of the magnetic nanoformulations at the tumour site, thus reducing any off-target effect on the tumour-surrounding healthy tissues. As shown in Figure 7b, the growth of 4T1 primary tumours was extensively suppressed by simultaneously promoting the activation of cytotoxic T cells and blocking the immunosuppressive PD-L1 pathway at the tumour microenvironment using the multifunctional IO@FuDex^3^ under magnetic navigation. Additionally, the IO@FuDex^3^ formulation showed to be also efficient for the treatment of CT26 colon cancer and lung metastasis in a 4T1 breast tumour model (Figure 7b,c). The antibodies’ conjugation onto IO@FuDex3 and magnetic navigation minimized the observed adverse events, and notably, were effective at extending the survival of treated mice with a dose more than 100 times inferior to soluble anti-PD-L1. Therapeutic approaches like the one described could potentially improve the therapeutic index of antibody-based immunotherapies, also allowing the development of combination therapies with reduced toxicities.

### 8.2. Immunotherapy in Combination with Other Cancer Therapies

Multimodal therapeutic strategies based on the combination of immunotherapy and other cancer therapies (e.g., chemotherapy and magnetic hyperthermia) have displayed synergistic effects that potentiate its efficacy, compared to single-based therapy, and have recently gained increased attention.

Chemotherapy is the most commonly utilized therapeutic modality to treat cancer in the clinic. Thus, the combination of immunotherapy and chemotherapy has been considered to develop strong collective anti-tumour effects. To integrate these two therapies into a single magnetic nanosystem, Hernández-Gil and co-workers reported magnetic micelles of phospholipids containing iron oxide nanoparticles to co-deliver anticancer platinum(IV) prodrug to induce tumour cell death, and TLR3 ligand poly(I:C) as an immunostimulant to activate DCs to promote protective anti-tumour immune responses [139]. The inert platinum(IV) prodrug became active as cisplatin in the highly reducing environment of the tumour site, exerting a cytotoxic effect against tumour cells. On the other side, poly(I:C) stimulated DCs by inducing TLR 3 signalling with a consequent increase in the production of IL-12. The secreted IL-12 mediated the activation of the NK cells and T cells, increasing their cytotoxic activity against malignant cells. The cytotoxic effect of cisplatin combined with the induction of both innate and adaptive immunity by poly(I:C) prevented tumour growth.

**Figure 8:**
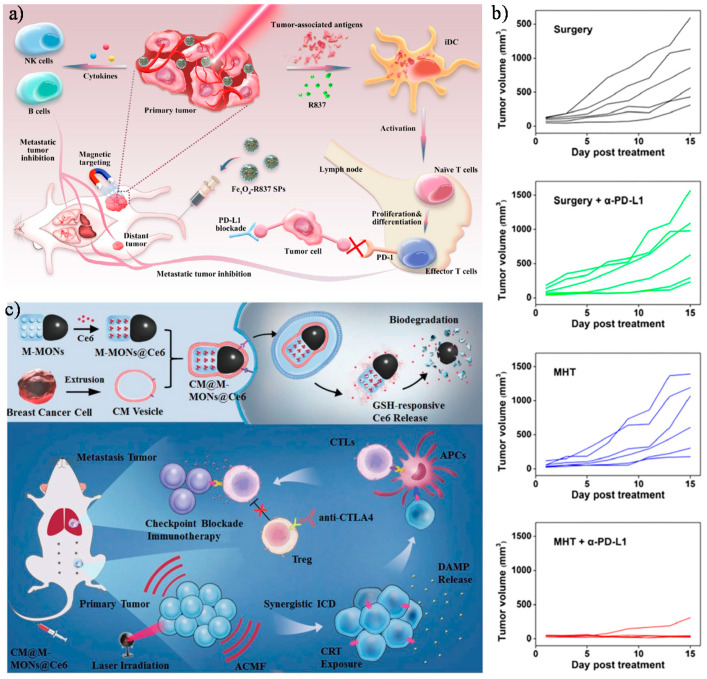
(**a**) Schematic demonstration of the synergistic anti-tumour effects of checkpoint blockade immunotherapy in combination with photothermal/R837-based therapy for promoting innate and adaptive anti-tumour responses. (**b**) Individual growth curves of mice divided into four treatment groups: surgery, surgery + α-PD-L1, MHT, and MHT + α-PD-L1. (**c**) Schematic representations of Janus magnetic mesoporous organosilica nanoparticles loaded with chlorine c6 and coated by cancer cell membrane for tumour targeted combined cancer therapy (top). Magnetic hyperthermia and PDT synergize in triggering ICD in cancer cells, by inducing the release/exposure of DAMPs and TAAs, responsible for the activation of APCs. Once activated, APCs prime effector CD8^+^ cytotoxic T cell responses. Co-administration of anti-CTLA4 antibody can avoid T-cells anergy induction for an enhanced anti-tumour immune response that can inhibit primary tumour growth and prevent lung tumour metastasis (bottom). Reproduced with permission from References [140,141,143] (Copyright 2018 and 2020 American Chemical Society and Copyright 2019 Wiley-VCH).

Photothermal therapy is a minimally invasive and promising therapeutic approach relying on the activation of photosensitizing agents by laser irradiation at near-infrared (NIR) to generate heat for the thermal ablation of tumours [145]. Photothermal therapy is shown to be effective at generating ICD and has been recently exploited in combination with immunotherapies in preclinical studies to overcome tumour resistance mechanisms [145]. Besides plasmonic materials, iron oxide nanoparticles absorb and efficiently convert heat infrared radiation at 800 nm into heat, making them interesting for photothermal applications [146]. For example, magnetically targeted photothermal immunotherapy of 4T1 triple-negative breast tumours was realized using nanoclusters of spherical iron oxide of 150 nm diameter with a high photothermal conversion efficiency of 68.2%. These magnetic nanoclusters contained self-assembled ultrasmall superparamagnetic iron oxide nanoparticles that act as photothermal agents under a laser excitation of 808 nm, and the synthetic TLR7-agonist imiquimod (R837) loaded into an amphiphilic polymer matrix of mPEG-PLGA [140]. The heating to 50 °C generated upon light irradiation with an NIR-laser of power density 0.33 W/cm^2^ for 30 min triggered a rapid release of the encapsulated R837 molecules at the tumour site and concomitantly promoted tumour cell elimination. The release of the TLR7 agonist together with the release of tumour-associated antigens (TAAs) by dying cancer cells led to DC maturation and the secretion of various cytokines (e.g., TNF-α and IL-6) (Figure 8a). Although the antitumor responses induced in the treated mice successfully inhibited the growth of primary orthotopic 4T1 tumours, they failed to protect from the spontaneous growth of the metastatic nodules in the lung and liver. To further improve the anti-tumour effect and, particularly, to treat/prevent metastatic disease, the authors combined the photothermal/R837-based treatment with intravenous injection of the PD-L1 antibody, resulting not only in primary tumour elimination but also in the prevention of the spontaneous growth of metastatic nodules in lungs and liver. This synergistic therapy also displayed abscopal effects that led to complete tumour growth inhibition of untreated distant tumours through the triggering of immune cell infiltration into the TME. Despite the induction of a strong systemic anti-tumour immune response, the combination treatment did not display signs of toxicity in the treated mice, thus indicating that strategies like the one proposed may be suitable for the development of safe and effective combinatorial immunotherapies.

Similarly, exploiting the heat generated by MNPs under radiofrequency excitation in the so called MHT has also shown a synergistic effect with cancer immunotherapies in preclinical studies. MHT may be particularly suitable for increasing the responsivity to checkpoint blockade therapies, as it has the potential to promote immune cell trafficking into tumours and, by inducing ICD, to expand the pool of antigen-specific T cells [141]. Its clinical implementation also may be favoured given that the accumulated MNPs into the tumour tissue generate heat locally, without affecting the adjacent healthy tissues and, importantly, in contrast to photothermal therapy, it particularly enables the treatment of deep-seated tumours, owing to the unlimited tissue penetration ability of the alternating magnetic field in MHT. Pan et al. recently reported magnetic hyperthermia therapy using dimercaptosuccinic acid-modified CoFe_2_O_4_@MnFe_2_O_4_ core–shell nanoparticles as a heat mediator with an SAR of 110 W/g at a condition of 577 kHz and 1.7 mT, in combination with checkpoint blockade immunotherapy for the elimination of both primary and metastatic tumours (Figure 8b) [141]. The authors demonstrated the excellent biocompatibility of the superparamagnetic nanoparticles up to a nanoparticle concentration of 400 µg/mL against both tumour and non-malignant cells. Although the conditions of the external alternating magnetic field used in this study are far from the clinical conditions of MHT (frequency, f, of 110 kHz and maximum field intensity, H, of 30 mT), this proof-of-principle study provided insights into the double therapeutic action of magnetic field-triggered heat. The heat generated by core–shell MNPs at the primary tumour site not only promoted direct tumour cell killing, but also induced a T cell-mediated anti-tumoural immune response that prevented the growth of distant tumours. Importantly this anti-tumour effect was enhanced when combined with anti-PD-L1 therapy to potentiate T-cell killing activity against tumour cells. Indeed, the therapeutic effect achieved by the combination treatment was superior in preventing primary and metastatic tumour growth compared to other therapeutic approaches, such as surgical resection alone or in combination with anti-PD-L1 treatment (Figure 8b). Future work may require conducting these studies with MNPs that enable heating by applying an external magnetic field that does not exceed the biological limit of H × f ≤ 5 × 10^9^ Am^−1^s^−1^, thus not raising concerns about the safety and clinical translatability of this strategy.

Recently, Wang et al. prepared multifunctional nanoparticles to combine photodynamic therapy (PDT) with magnetic hyperthermia (MHT) to synergistically improve the immunogenic capacity of dying cancer cells to elicit anti-tumour immune responses (Figure 8c) [143]. The authors developed bullet-shaped Janus magnetic mesoporous organosilica nanoparticles (M-MONs@Ce6) composed of iron oxide nanoparticles placed on the head of the Janus particle used as a heat mediator for MHT and having the body of the disulphide-bridged mesoporous organosilica, into which the most commonly used photosensitizer for PDT, chlorine e6 (Ce6), was incorporated. Next, in order to improve the colloidal stability in physiological environments and to attain the homologous tumour targeted accumulation of M-MONs@Ce6, each nanoparticle was further entirely coated with breast cancer cell-derived membrane. The biodegradability of the nanostructures made them highly responsive to redox/pH variations, thus ensuring the precise release of the photosensitizer over time in the acidic and reductive conditions of TME (Figure 8c). Furthermore, the application of an alternating current magnetic field (32.5mT, 262 kHz) for 20 min prior to PDT not only destroyed the tumour cells but also improved the tumour oxygenation via promoting blood vessel damage, which is more beneficial for PDT in the hypoxic regions of tumours. Therefore, under irradiation of 606 nm light (0.15 W/cm^2^, 10 min), the released chlorine e6 at the tumour had a high capability in the enhancement of intracellular reactive oxygen species, which was sufficient to eradicate cancer cells. Consequently, after the combined application of the treatments, primary breast tumour growth was strongly inhibited and this outcome correlated with profound changes in the TME, including increased number of cytotoxic T cells and decreased frequency of Tregs [143]. This immune response was further amplified by combination with anti-CTLA-4 antibody, thus suppressing the growth of both primary and metastatic tumours [143].

## 9. Conclusions and Future Perspectives

Despite the high potential of cancer immunotherapy, its clinical application in a larger range of tumour settings is still pending. Toxicity and therapeutic responsivity restricted to a small fraction of patients represent the major challenges. The variability observed in patients’ response rates reflects the several different pathways utilized by tumours to control the multiple mechanisms taking place in the TME to evade immune responses. Hence, immunotherapy aimed to target a single specific protumoral mechanism appears to be inefficient at achieving a significant therapeutic effect. To guarantee the development of novel effective cancer treatments, the combination of therapeutic strategies that simultaneously hit various mechanisms underlying cancer immuno-evasion is highly desirable, although this may be associated with increased toxicity. In recent years, nanoparticle-based delivery systems have demonstrated great potential to ameliorate the effectiveness and safety profile of conventional immunotherapeutics, acting as vehicles for the precise delivery of tumour antigens and/or immunostimulatory molecules to specific cells located in lymphoid organs or in the TME. In particular, MNPs are gaining significant attention because of their unique capability to respond specifically to an applied external magnetic field, which is particularly interesting for biomedical applications and has enabled the development of novel immunotherapeutic approaches relying on heating capability, magnetically controlled navigation, and image-guided strategies, such as MRI and MPI.

Magnetic heating-based therapy has been exploited as an ICD inducer, demonstrating great suitability for the implementation of in situ vaccination strategies that do not require previous identification and selection of tumour antigens. Furthermore, the use of magnetic nanostructured systems has facilitated the combination of MHT with immunotherapeutic approaches, such as checkpoint inhibitors, increasing their response rates, reducing drug resistance, and, remarkably, minimizing the therapeutic doses. These kinds of combinatorial treatments have been possible thanks to the development of multicomponent nanocarriers that can integrate diverse functions into a single nanosystem. Importantly, MHT technology has already been translated into the clinic for the treatment of recurrent glioblastoma and prostate cancer by MagForce, demonstrating the clinical feasibility of MHT strategies. Moreover, many nanoformulated iron oxide-based complexes have been FDA-approved for use as contrast agents in MRI (i.e., Resovist, Feraheme, Feridex, Clariscanand VSOP C184) or as iron supplements for patients with iron deficiency (i.e., Ferinject and Verofer), or as food additives (i.e., E172), suggesting their biocompatibility.

Although several MHT-based combinatorial approaches have been largely investigated in preclinical stages, their translation into the clinic is hindered due to the absence of satisfying data regarding the safety of the proposed nanoformulations. Certainly, the use of materials that are FDA-approved or for which more toxicity information is available might speed up the introduction of magnetic multicomponent nanosystems for combinatorial strategies into clinical trials. PLGA and lipidic nanoparticles represent some of the highly biocompatible nanomaterials that have been recently employed for the development of multifunctional magnetic nanostructures, the use of which may be suitable for enabling the design of MHT combinatorial immunotherapies that could be applied in the clinic. Immunotherapy combined with MHT could also represent a valid approach to overcome the existing limitations of MHT.

Magnetic navigation/image guidance represents another promising area of application of MNPs, which can enable precise delivery of both immune cells and immunotherapeutics. Magnetic navigation has been successfully applied to guide drugs and cell therapies at the target region in preclinical studies, allowing to improve their therapeutic efficacy and minimize toxicity and the required dosage. All these can be relevant for ICB therapy, in which one of the main limitations currently involves the toxicity associated with the broad blockage of checkpoint pathways, and the high cost per dose. Likewise, the application of magnetic navigation/image guidance for ACT may be a breakthrough, as it could potentially facilitate the accumulation of lymphocytes at a desired site, and thus their therapeutic activity. Despite this potential, the applicability of magnetic navigation strategies to boost immunotherapies in clinical settings remains challenging. Indeed, it is still yet to be proven if new technologies will be suitable for the generation of a magnetic gradient that can achieve the spatial–temporal localization of MNPs at deep-seated tumours.

The image-guided approach has emerged as a potential promising technique to enable simultaneous tumour localization and treatment with great precision. In particular, MPI-based treatments are gaining a growing interest, offering superior clinical performance compared to MRI since they display a higher sensitivity and signal-to-noise ratio. Preclinical studies have shown that immune cells (e.g., NK cells) can be loaded with iron oxide nanoparticles without compromising their functions and used as MPI tracer or magnetic navigation to increase delivery of nanoparticles at the desired site. In these types of approaches, iron oxide nanoparticles can serve both as tracers in MPI and MHT-mediators since they can be excited through an external magnetic field to generate heat. Potentially, this combination may enable to extend conventional MHT, in which iron nanoparticles are deposited intratumorally and then deliver heating locally, to the possibility to image and precisely select the tumour area, thereby preventing off-target treatments of the liver and spleen, organs in which MNPs tend to accumulate.

To conclude, magnetic nanosystems hold tremendous potential for safe, more effective, and personalized cancer treatment, allowing a localized delivery of payload drugs and facilitating the rational design of novel combinatorial therapies based on immunotherapeutic treatments, exploiting the adaptive and/or the innate immune system, such as ACT, therapeutic vaccines, and immunomodulatory therapies. Although in the initial phase of its development, magnetic-guided immunotherapy represents an additional tool that could also help to advance the field of cancer immunotherapy. Future studies aimed to overcome the current technical limitations of magnetic field-generating equipment and to improve the magnetic properties of magnetic nanomediators could help expand the use and clinical implementation of magnetic-responsive nanosystems.

## Figures and Tables

**Table 1:** Overview of the different magnetic nanostructure-based combinatorial immunotherapy approaches.

Magnetic Nanostructure	Surface Chemistry	Immunotherapeutic Drug	Therapeutic Approach	Remarks	Ref.
Iron nanoparticles (nano-aAPC)	Dextran functionalized with both MHC-Ig dimer and anti-CD28 antibody	MHC-Ig dimer, anti-CD28 antibody	Adoptive immunotherapy	Application of an external magnetic field induced nano-aAPC aggregation on naive cells, enhancing T cell proliferation in vitro and following adoptive transfer in vivo.	[60]
Iron oxide nanoclusters (Magnetosome)	Cancer cell-derived plasma membrane functionalized with anti-CD205 antibody	TAAs, CpG ODN	Vaccine/Immune adjuvant	Cancer cell membranes serve as a reservoir of TAAs and their co-delivery with TLR9-agonist lead to a great proliferation of T-cells with superior cytotoxic activity. The application of an external magnet enhanced lymph node retention and anti-CD205-mediated CD8^+^ DCs targeting of nanoparticles.	[59]
Iron oxide nanoclusters (IO-LPMONs)	Mesoporous organosilica shell having large pore size	OVA antigen	Vaccine/TAMs repolarization	Simultaneous T cell activation and TAMs repolarization induced strong inhibition of tumour growth.	[138]
Iron oxide nanospheres (IO@FuDex^3^)	Fucoidan and dextran functionalized with multiple antibodies	Anti-PD-L1, anti-CD3 and anti-CD28 antibodies	T cell activation/Immune checkpoint inhibitor	IO@FuDex^3^ can directly induce T-cell activation and block the immunosuppressive PD-L1 pathways via intravenous administration. The combination of IO@FuDex^3^ and magnetic navigation demonstrated a highly improved therapeutic efficacy.	[116]
Iron oxide nanoparticle-loaded micelles (poly(I:C)–Pt(IV)–IONP micelles)	DSPE-PEG(2000)-Pt(IV) prodrug functionalized with poly(I:C)	Poly(I:C)	Immune adjuvant/Chemotherapy	Pt(IV) prodrug synergized with TLR3-agonist inducing a more potent activation of DCs than cisplatin and poly(I:C).	[139]
Iron oxide superparticles (Fe_3_O_4_-R837 SPs)	Poly(ethylene glycol)-block-poly(lactic-co-glycolic acid) copolymer	R837, anti-PD-L1 antibody	ICD/Immune adjuvant/Immune checkpoint inhibitor	Photothermal therapy promotes cancer cells killing, with consequent release of TAAs, and triggers the release of R837 immune adjuvant for a more effective vaccination strategy. Fe_3_O_4_-R837 SPs efficiently synergize with PD-L1 antibody to eliminate the primary tumours and prevent tumour metastasis to lungs/liver.	[140]
Core-shell ferrite nanoparticles (CoFe_2_O_4_@MnFe_2_O_4_ nanoparticles)	Dimercaptosuccinic acid molecule	Anti-PD-L1 antibody	ICD/Immune checkpoint inhibitor	Magnetic hyperthermia induced TAAs release eliciting a systemic immune response affecting distant metastatic tumours. The combined MHT and checkpoint inhibitor demonstrate the great potentials in inhibiting the growth of both primary and metastatic tumours.	[141]
FePt/MoS_2_-FA nanocomposites (FPMF NCs)	FePt capped by dimercaptosuccinic acid, MoS_2_ modified by thiol-polyethylene glycol-folate	CpG ODN, anti-CTLA-4 antibody	ICD/Immune checkpoint inhibitor	PDT act as ICD inducer and its ability to inhibit primary tumours and prevent metastasis was significantly improved when combined with chemotherapy drug/immunotherapeutics.	[142]
Janus nanobullets integrating chlorine e6 (Ce6) loaded, disulfide-bridged mesoporous organosilica bodies with magnetic heads(M-MONs@Ce6)	Asymmetric mesoporous silica growth, coated with cancer cell membrane	Anti-CTLA-4 antibody	ICD/Immune checkpoint inhibitor	The combination of PDT and magnetic hyperthermia elicits ICD, resulting in tumour-specific immune responses. When combined with anti-CTLA-4 antibody, synergistically enables the eradication of primary and deeply metastatic tumours.	[143]
Iron nanoparticles (FeNPs)	Poly(acrylic acid) (PAA) co-grafted with dopamine (DA) and amine-terminated PEG (5 kDa)	R837	ICD/Immune adjuvant/Immune checkpoint inhibitor	The combination of MNP-based MHT with local injection of nanoformulated TLR7-agonist and systemic injection of anti-CTLA4 antibody resulted in systemic immune responses that inhibited tumour metastasis and recurrence.	[144]

## Abbreviations

ACT	Adoptive cell therapy
ADCC	Antibody-dependent cell cytotoxicity
APCs	Antigen-presenting cells
AMF	Alternating magnetic field
αβ T	Alpha beta T
BCR	B cell receptor
BNF	Bionized nanoferrite
CARs	Chimeric antigen receptors
CAR-T	Chimeric antigen receptor-T cell
CpG ODN	CpG oligodeoxynucleotides
CTLA-4	Cytotoxic T-lymphocyte antigen-4
DAMPs	Damage-associated molecular patterns
DCs	Dendritic cells
DNA	Deoxyribonucleic acid
ECM	Extracellular matrix
EGFR	Epidermal growth factor receptor
EPR	Enhanced permeability and retention
FDA	Food and Drug Administration
GM-CSF	Granulocyte–macrophage colony-stimulating factor
γδ T	Gamma delta T
HMGB1	High-mobility group box 1
HSP	Heat-shock protein
ICB	Immune checkpoint blockade
ICD	Immunogenic cell death
IFN-γ	Interferon gamma
IL	Interleukin
MDSCs	Myeloid-derived suppressor cells
MHC	Major histocompatibility complex
MHT	Magnetic hyperthermia therapy
MNPs	Magnetic nanoparticles
MPI	Magnetic particle imaging
mPEG-PLGA	Poly(ethylene glycol)-block-poly(lactic-co-glycolic acid)
MRI	Magnetic resonance imaging
NF-κB	Nuclear factor-κB
NIR	Near infrared
NK	Natural killer
OVA	Ovalbumin
PAMPs	Pathogen-associated molecular patterns
PBLs	Peripheral blood lymphocytes
PDA	Polydopamine
pDNA	Plasmid deoxyribonucleic acid
PD-1	Programmed cell death protein 1
PD-L1	Programmed death-ligand 1
PD	Photodynamic therapy
PEI	Polyethyleneimine
poly(I:C)	Polyinosinic:polycytidylic acid
PRRs	Pattern recognition receptors
RNA	Ribonucleic acid
siRNA	Small interfering ribonucleic acid
SAR	Specific absorption rate
SPs	Superparticles
SPION	Superparamagnetic iron oxide nanoparticle
TAAs	Tumour-associated antigens
TAMs	Tumour-associated macrophages
TCR	T-cell receptor
TILs	Tumour-infiltrating lymphocytes
TME	Tumour microenvironment
TNF-α	Tumour necrosis factor alpha
TGF-β	Transforming growth factor-beta
Th	T helper
TLR	Toll-like receptor
Tregs	Regulatory T-cells
